# Clinical correlates of plasma ghrelin and LEAP-2 concentrations in Eating Disorders and Obesity

**DOI:** 10.1192/j.eurpsy.2025.700

**Published:** 2025-08-26

**Authors:** I. Baenas, L. Camacho-Barcia, R. Granero, S. Tamarit, S. Jiménez-Murcia, F. Fernández-Aranda

**Affiliations:** 1 Clinical Psychology, Bellvitge University Hospital; 2 Eating and Addictive Behaviours, Neuroscience Program, Bellvitge Biomedical Research Institute (IDIBELL); 3 CIBER Physiopathology of Obesity and Nutrition (CIBERobn), Instituto Salud Carlos III; 4 Doctoral Program in Medicine and Translational Research, University of Barcelona; 5 Psychobiology and Methodology, Autonomous University of Barcelona; 6 University of Barcelona, Barcelona, Spain; 7 Clinical Sciences, School of Medicine and Health Sciences; 8 Psychology Services, University of Barcelona, Barcelona, Spain

## Abstract

**Introduction:**

Hormones such as ghrelin and the recent described human liver expressed antimicrobial peptide-2 (LEAP-2) play a pivotal role in regulating food intake and energy metabolism. In this context, their aberrant functioning has been suggested as a mechanism underlying the pathophysiology of eating disorders (ED) and obesity. Considering the high comorbidity between binge spectrum disorders (BSD) and obesity, whether this dysfunction could be a potential shared neurobiological mechanism remained underexplored.

**Objectives:**

To analyze plasma ghrelin and liver expressed antimicrobial peptide-2 (LEAP-2) concentrations in fasting among individuals with obesity (OB), with and without an eating disorder (ED), and compare them with a group of healthy controls (HC). Besides, to assess associations between these concentrations, psychopathological variables, and body mass index (BMI) in the clinical sample with OB.

**Methods:**

The sample comprised 162 adult women (67 OB-ED, 35 OB+ED, and 62 HC). Peripheral blood samples, eating psychopathology, trait impulsivity, food addiction (FA), fat mass, and BMI were collected. The between-group comparisons were performed using analyses of covariance (ANCOVA), adjusting for age and fat mass, and the link between variables was evaluated through correlation and path analyses.

**Results:**

Individuals with OB (with and without an ED) showed lower significant ghrelin concentrations than HC (p<.001). The OB+ED group reported significantly higher eating psychopathology, trait impulsivity, and FA than the OB-ED and HC groups. In the OB+ED group, LEAP-2 concentrations positively correlated with BMI, fat mass, novelty seeking, and FA scores. The path analysis showed that higher LEAP-2 levels and FA scores were linked to more severe eating psychopathology

**Conclusions:**

The results suggest an interplay between biological and clinical factors that contribute to delineate vulnerability pathways in ED and OB, which could help fit more tailored therapeutic approaches.

**Disclosure of Interest:**

None Declared

